# Towards Achieving the Full Clinical Potential of Proton Therapy by Inclusion of LET and RBE Models

**DOI:** 10.3390/cancers7010460

**Published:** 2015-03-17

**Authors:** Bleddyn Jones

**Affiliations:** Gray Laboratory, CRUK/MRC Oxford Oncology Institute, The University of Oxford, ORCRB-Roosevelt Drive, Oxford OX3 7DQ, UK; E-Mail: Bleddyn.Jones@oncology.ox.ac.uk; Tel.: +44-792-661-7060; Fax: +44-792-661-7334

**Keywords:** proton therapy, radiotherapy, RBE, mathematical model, radiobiology

## Abstract

Despite increasing use of proton therapy (PBT), several systematic literature reviews show limited gains in clinical outcomes, with publications mostly devoted to recent technical developments. The lack of randomised control studies has also hampered progress in the acceptance of PBT by many oncologists and policy makers. There remain two important uncertainties associated with PBT, namely: (1) accuracy and reproducibility of Bragg peak position (BPP); and (2) imprecise knowledge of the relative biological effect (RBE) for different tissues and tumours, and at different doses. Incorrect BPP will change dose, linear energy transfer (LET) and RBE, with risks of reduced tumour control and enhanced toxicity. These interrelationships are discussed qualitatively with respect to the ICRU target volume definitions. The internationally accepted proton RBE of 1.1 was based on assays and dose ranges unlikely to reveal the complete range of RBE in the human body. RBE values are not known for human (or animal) brain, spine, kidney, liver, intestine, *etc.* A simple efficiency model for estimating proton RBE values is described, based on data of Belli *et al.* and other authors, which allows linear increases in α and β with LET, with a gradient estimated using a saturation model from the low LET α and β radiosensitivity parameter input values, and decreasing RBE with increasing dose. To improve outcomes, 3-D dose-LET-RBE and bio-effectiveness maps are required. Validation experiments are indicated in relevant tissues. Randomised clinical studies that test the invariant 1.1 RBE allocation against higher values in late reacting tissues, and lower tumour RBE values in the case of radiosensitive tumours, are also indicated.

## 1. Introduction

Radiotherapy has evolved empirically, with progressive technical improvements, most of which have contributed to better clinical outcomes as either a better cure rate, or reduced complication rate, or both. Early and late tissue side effects are important and do frequently limit presently prescribed doses, as also may changes in tissue radiation tolerances due to surgery, concomitant medical conditions, and various forms of chemotherapy. Intensity modulated photon radiotherapy (IMRT) is a modern computerised development of the use of wedge filters and tissue compensators in the 1940s by Frank Ellis [1]. Changes in beam fluence along the width of an IMRT beam has allowed critical organs to be spared, but with fluence transfer to other organs and tissues considered to be less critical. With increasing recognition that late vascular damage is responsible for many chronic effects of radiation and that the relative risk of radiation induced coronary artery disease is estimated to be around 7.5% per Gray [2], as well as the established cancer induction risks of at least 5% per Sievert [3], leads to a logical requirement for radiation beams that can markedly reduce integral dose and so the amount of tissue traversed. This can be achieved with positively charged particle beams such as protons and light ions, due to their reduced path length in tissues caused by the Bragg peak (BP) effect.

Technical developments in radiotherapy need careful assessment of their biological effects, since intended gains can occur along with subtle or even major disadvantages. In the case of proton beam therapy (PBT), despite its increasing use concerns remained about the lack of published evidence showing clear cut improvements in outcomes. Many overviews point out the paucity of publications many of which show no clear-cut improvements in terms of tumour control and/or late normal tissue complications, as well as the lack of high level medical evidence using randomised studies [4,5,6,7,8]. The various authors have emphasized that most publications concerned with PBT provide dose distribution data and consider technical advances.

PBT, despite its attractive and well-known ballistic features, has two intrinsic (but not insuperable) weaknesses:

- accuracy and reproducibility of BP position.
- inadequate knowledge of the relative biological effect (RBE) in different tissues and at different doses.

**Figure 1:**
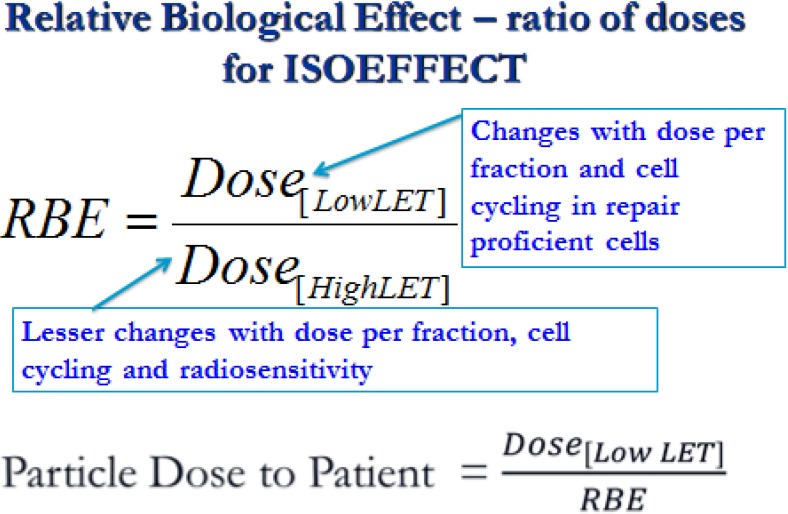
Definition of relative biological effect (RBE).

To understand these, it is necessary to consider ionisation patterns. The linear intensity of energy release on a microscopical scale, and independent of volumetric dose, is expressed as linear energy transfer (LET). Increasing LET, above that experienced in conventional photon (x-ray) therapy, occurs mostly within BP regions. Since BPs are spread out to cover a defined clinical target volume, thus mixing BP and non-BP regions, the LET increases can be relatively modest, but will always cause increased cellular radiosensitivities. In higher LET regions there is increased clustering of DNA damage, which is less amenable to repair by cellular enzyme systems, resulting in greater complexity of lethal chromosomal aberrations [9]. Increased radiosensitivities lead to a modification of dose required to achieve the same biological effect, and is measured as the relative biological effect (RBE). RBE is of paramount importance since it determines the dose given to the patient in Equivalent-Gy as shown in Figure 1. Essentially, if the RBE is incorrect the proton dose may be too low or too high, with consequent effects in each patient [10].

RBE depends on multiple factors: LET, beam energy, particle nuclear charge (z), target volume and depth, as well as multiple biological factors that influence DNA repair capacity and radiosensitivity in different cellular types and tissues [11,12,13,14,15,16]. This multifactorial basis of RBE implies that it cannot be a constant value, but must be a continuous variable (as is height and weight in humans). Particularly relevant are the radiobiological properties of the control low LET of conventional megavoltage radiotherapy which govern the numerator of the RBE definition (see Figure 1). From the shapes of the cell survival curves at low and high LET [11,12,13,14,15,16], it can be seen that the numerator dose will increase to a greater extent than the denominator dose for a given iso-effect when dose per fraction is changed.

This report considers some aspects of proton range uncertainties, and RBE issues in greater detail, along with description of a new RBE predictive model.

### 1.1. Proton Range Uncertainties

The energy spread of protons, their interactions in non-homogenous tissue, as well as physiological movement of tissues, all contribute to range uncertainties, and so to Bragg peak positions (Paganetti [17,18]). These uncertainties will vary, depending on anatomical site, from millimetres to several centimetres (in the case of physiological movements). Patient positioning variations, gain or loss of weight during treatment, with resultant changes in subcutaneous fat depths, tumour shrinkage, can all critically affect particle range, dose and LET distribution, with implications for tumour control and toxicities. Imaging of the BP position is improving, for example with proton activated PET signal detection to an accuracy of 2 mm [19].

**Figure 2:**
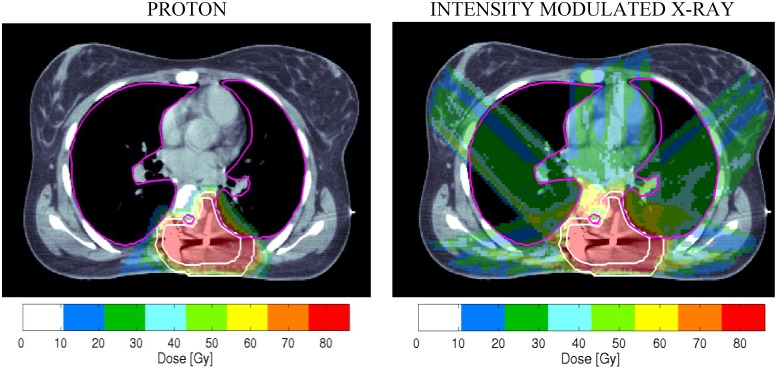
Some clinical dilemmas in particle therapy are shown in these dose distributions displayed on a CT scan of a female patient who has had incomplete surgery for a paravertebral epithelioid sarcoma. (Plan obtained with permission of J. Munzenrider, Massachusetts General Hospital).

A clinical example of some of these considerations, and RBE allocation uncertainties, are shown in Figure 2. The proton “dose” shown is calculated using an RBE conversion factor of 1.1. (the equivalent x-ray dose is divided by 1.1). The required 76 Gy dose to the target volume is achieved in both cases, but the proton plan spares the heart, a considerable volume of lung, and breast tissue, so reducing the later risk of breathlessness on exertion, sudden cardiac death and of radiation induced breast cancer. The small ringed area anterior to the high dose volume shows the position of the spinal cord and the dose received to this structure appears to be same for both treatment plans.

Despite these attractive features, what if the RBE of 1.1 used in the dose prescription is higher than that of the tumour and lower than that of the spinal cord? Then, there would be enhanced risks of both tumour recurrence and spinal damage (resulting in paralysis of the lower limbs) when compared with the x-ray based treatment. Also the effect of increasing particle range, as would happen with loss of weight, might lead to a higher LET and dose in the spinal cord, increasing the risk of paralysis, while also lowering LET and dose to the tumour, so reducing the probability of tumour control. The simplest allowance for changes in tissue distances would be to allow millimetre exchanges, assuming uniform tissue density. In this way each millimetre lost or gained will shift the high dose boundary in either direction by the same amount. The ballistic advantages, changes in dose distribution and these more uncertain aspects of PBT should be mentioned in informed consent procedures to patients, with explanation of how these risks can be minimised.

The example in Figure 2 illustrates the interdependence of dose, LET and RBE on the potential clinical outcomes. Further discussion of RBE is given below.

To reduce range instabilities, meticulous daily assessment of patient dimensions, accurate tumour localisation and patient immobilisation *etc*., including confirmatory measurements made by non-ionising techniques such as ultrasonography, are necessary requirements. Improved dose computing techniques and 4-D (time-included) planning, including adaptive techniques to changes in normal tissues and tumour positions/dimensions with time are even more important in PBT than conventional radiotherapy. Recent work by Schuemann *et al.* [18] considers the accuracy of obtaining the physician defined target volume margins and provides recommended tolerances for different anatomical sites. A recent review of Proton radiography and PET, or prompt gamma ray imaging, using small test doses of radiation or actual treatments respectively may also improve overall quality assurance [19,20,21,22]. The inaccuracies associated with algorithms in proton planning, which can vary with anatomical site, can be improved by application of Monte Carlo planning methods [17,18]. All these advances must be interpreted alongside the difficulty in establishing the true extent of tumour infiltration into normal tissues since present imaging methods do provide the necessary resolution to detect small clusters or individual cancer cells.

There has always been concern that the highest LET and RBE will occur towards the end of the particle range. This led to “patch field” techniques, which aligns lateral beam edges against sensitive anatomical structures, when using passive scattering [23]. In contrast, lateral beam edge beam uncertainties are not so well understood. Beams diverge with depth due to Coulomb scattering, as well as geometric divergence with passively scattered beams. Lateral scatter must carry implications for higher LET values, since deviated particles will form Bragg peaks in a more lateral direction. Also the tracks become more separated and less linear, so a more micro-volumetric assessment of energy transfer (MVET) is indicated, as well as the number of particles per unit volume. There is already evidence that with spot scanned intensity modulated proton therapy high LET values can be found outside the tumour bearing target volumes [24]. This is perhaps explained by a greater proportion of partially overlapping beam edges, along with the additional weighting applied to some beamlets.

### 1.2. RBE Uncertainties

The RBE concept is so often either misunderstood or underestimated in terms of complexity. It is vital to understand its multifactorial dependency, already listed above. All clinicians should understand that if the RBE is incorrect, so also will the dose given to the patient, sometimes by a percentage change that can exceed normally acceptable treatment plan dose variations, or the legally permitted range of dose due to errors in beam delivery [10]. RBE also has an impact on proton range uncertainties [25]. A reported reduction in RBE at long range [26] is possibly connected with the MVET of increasingly separating tracks, and has important implications which need further experimentation.

Proton RBEs have similarities with those for heavier ions than protons, but with some differences in scale. High proton RBEs have been found in various experiments, in high LET parts of the beam [11,12,27,28,29]. Also, the rise in RBE per unit increase in LET is larger at lower LET values for protons compared to all heavier ions. This causes the turnover of RBE with LET at around 30.5, rather than at between 100–120 and 180–220 keV·μm^−1^ for protons, helium and carbon ions respectively [11,12,13,14,15,16]. Thus a greater respect for potential high RBE values of protons is required.

The essential clinical features of RBE are its inevitable dependency on LET, dose and tumour/tissue type. Changes in proton RBE with dose per fraction and tissue type have not been as extensively researched when compared with the range of experimental systems used for fast neutrons. In the latter case, there was a clear dependency on tissue type: fast proliferating, acute reacting tissues (with high α/β ratios) had much smaller changes in RBE with dose than were the case for far more slowly proliferating late reacting tissues (with low α/β ratios) [27]. The collated proton experiments, and the expedient decision to allocate an overall RBE of 1.1, representing a median value, to all tissues and tumours (regardless of their α/β ratio), summarised by Paganetti *et al*. [28,30], must be reconsidered for the following reasons. Fast growing assays were used, with a preponderance of high dose exposures between 9–12 Gy for *in vivo* later tissue reactions (only in lens of eye, skin and lung), where only low RBE values can be expected. These high doses reflected clinical practice in therapy of uveal melanomas, but much lower doses (1–2 Gy) are used in other anatomical sites. There needs to be a comprehensive re-assessment of proton RBE, using more relevant longer-term assays and more typical dose ranges. This should be coupled with more detailed analysis of human clinical outcomes, inclusive of potential LET-RBE effects, as discussed further below.

## 2. Methods

### 2.1. Description of Quantitative Model

**Figure 3:**
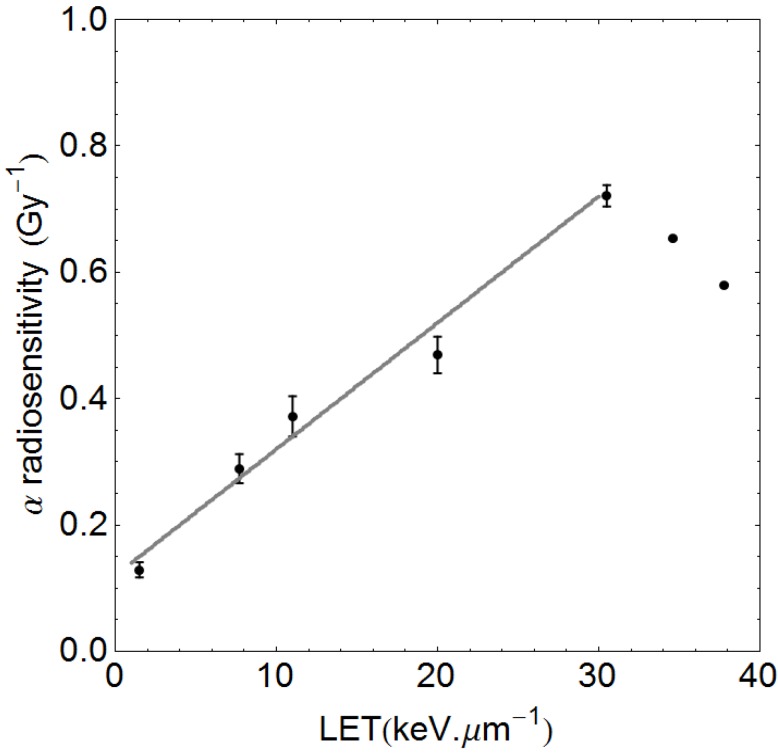
Data of Belli *et al*. [11] showing the linear rise in α with linear energy transfer (LET), which is linear and can be fitted up to the turnover point. The least squares fitted line is α_H_ = 0.12 + 0.02 LET, which will be unique for the V-79 cells used, where 0.12 Gy^−1^ is the α_L_ value for the control radiation. The two data points beyond the turnover point are not used in the pre-turnover point slope determination.

The experiments of Belli *et al.* [11] have shown that proton LET and RBE rise sharply at much lower values of LET than is the case for other heavier particles. The maximum efficiency is at just over 30 keV·µm^−1^, where there is a turnover point (see Figure 3), after which overkill occurs due to ‘wasted dose’ at the genetic target level: more than one lethal break in the same chromosome will not produce additional cell kill despite the enhanced energy deposition. Conveniently, it is not necessary to consider values near to or beyond the turnover point since clinical treatment plans contain values between 1.5 and 9 keV·µm^−1^ due to spreading out of Bragg peaks [24]. The initial approach taken is similar to the useful publication by Wilkens and Oelfke [31], but with later differences. It is well established that the initial slope of the α radiosensitivity and RBE increments with LET are linear [32], although this is often masked when plotted on a logarithmic LET scale. The pre-turnover point α-radiosensitivity data of Belli *et al.*, taken from their Table 2 are plotted in Figure 3, where it can be seen that α increases linearly up to the turnover point. The same data show unexpected reductions in β values with increasing LET, probably linked to the small number of data points, the V-79 cell system used which tends not to show significant β increments, and the linear-quadratic fitting process as discussed elsewhere [33], and so cannot be regarded as reliable. Further information on β changes with LET are given in the Appendix.

**Figure A1:**
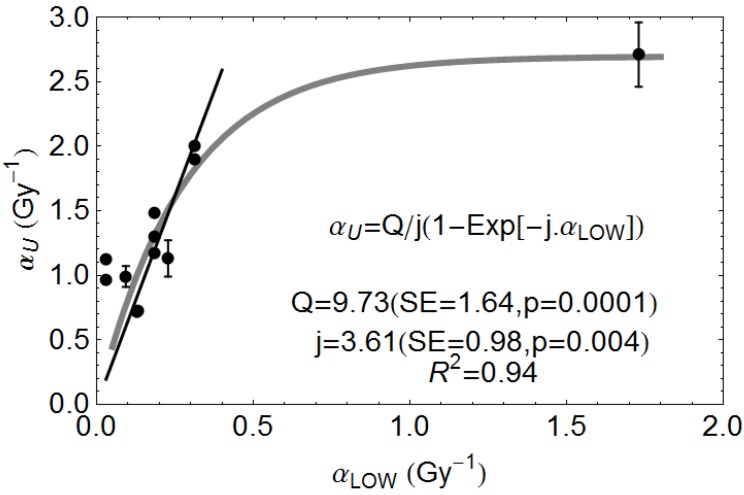
Plot the maximum α value at the LET-RBE turnover points and the control low LET α value for ion beam data [11,12,13,14,15,16]. Two least squares fits (linear and non-linear) are shown, each applicable for different ranges of the control α_L_ value. The linear fit is given by α_U_ = 6.47α_L_ (*p* < 0.001, R^2^ = 0.899) but only for α_L_ values up to 0.35 Gy^−1^.

The efficiency of cell killing, in terms of the α and β parameters, are both assumed to rise linearly with LET to a maximum at 30.5 keV·µm^−1^ and to a level determined by the low LET (control radiation) α and β parameters (respectively termed α_L_ and β_L_, but where α/β is used it will refer to the low LET state). The full proton RBE model is presented in the Appendix, and is generalised for any cell system with unique low LET radiosensitivities α_L_ and β_L_ as input parameters. This is possible since data from Belli, Furusawa, Barendsen, Weyrather *et al*. [11,12,13,14,15,16] on a variety of ions show a relationship between α_L_ and the maximum value of α (termed α_U_) at the LET-RBE turnover points (where LET is LET_U_), as shown as Figure A1 in Appendix. The estimated α_U_ value will determine the slope of α with LET. It would be inappropriate to use the slope found in the Belli *et al.* data for all cells and tissues, since different cell types will possess their own unique RBEs and α increments with LET, but share the same LET_U_ value [15,16], which is a characteristic for each ion species. The LQ model is then used to estimate iso-effective RBE for any dose per fraction (see Appendix).

### 2.2. The Qualitative ICRU Approach

Logical conditional statements are superimposed on the recommended ICRU treatment volumes [34], which are used in the treatment planning process. In this way, what can or cannot be achieved with PBT compared to best available megavoltage treatment (MXT) can be expressed in a simple matrix of conditional statements, enhanced by a further concept of tissue outside the target volume (referred to as the OTV), Normal tissues within the CTV and PTV are assumed to receive the same dose as any tumour cells. Inevitably, these expected results will be distorted by errors of dose placement and inappropriate RBE allocations, which can be expressed as inequalities.

## 3. Results

### 3.1. Quantitative Model Approach

**Figure 4:**
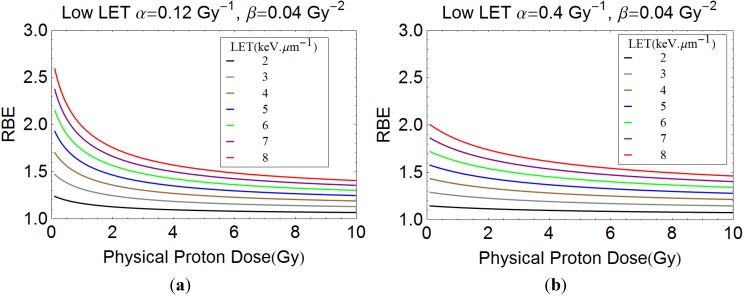
(**a**) and (**b**) show plots of RBE with dose per fraction for a wide range of LET values, with the assumed radiosensitivity parameters shown.

**Figure 5:**
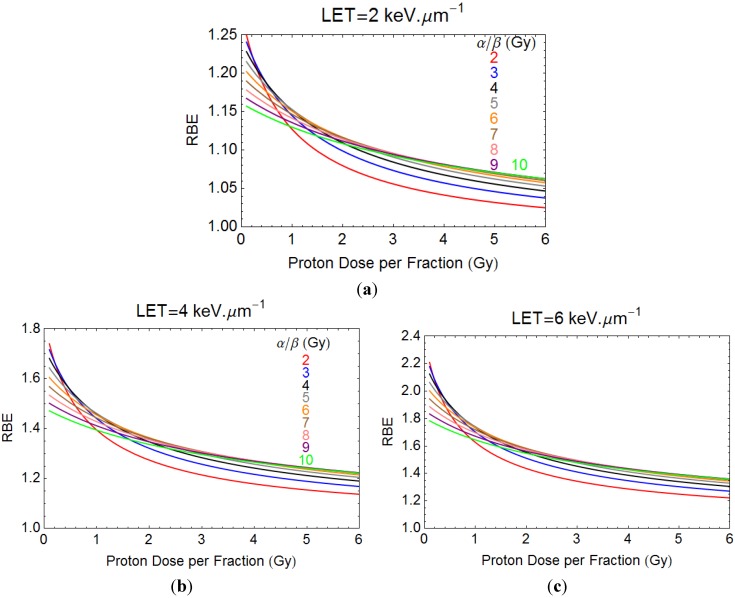
(**a**–**c**) show plots of RBE with dose per fraction for LET values of 2, 4 and 6 keV·µm^−1^.

For the quantitative modelling approach, plots of proton RBE with dose per fraction for different LET values are shown in Figure 4a,b for two examples of different radiosensitivities. These estimates show increasing RBE with LET and an inverse relationship between RBE and dose per fraction. Greater complexity is seen for variation of α/β ratios, as in Figure 5a–c. It again can be seen that increases in LET can be accompanied by significant increments in RBE. An RBE value of 1.1 covers most eventualities at a dose of 2 Gy only when the LET is 2 keV·µm^−1^. The lowest α/β ratio systems give the largest RBE at near zero dose, but then the values fall sharply, the rate of change depending on α/β. High α/β ratio systems have much flatter curves, showing less variation in RBE with dose per fraction. The curves cross over such that larger RBE values can exist for systems with high α/β ratios compared with low α/β ratios when dose per fraction is sufficiently large; it is important to note that the lowest RBE values then occur in the most fraction-sensitive tissues, suggesting that high dose per fraction can be relatively protective. This is because of enhanced repair capacity and fraction sensitivity in the case of low α/β ratios for the photon (numerator) part of the RBE definition and the converse for the high LET denominator dose. Also, cells with low α/β, with the highest RBEmax, will asymptotically approach RBE min with dose at a faster rate owing to the higher proportion of β related cell killing than in cells where α/β is high.

Many of the RBE values shown exceed the conventional value of 1.1. Of particular concern is that for low α/β ratio systems such as brain spinal cord and late reactions, the RBE values are highest when dose per fraction is small. This finding represents a warning to clinicians, who should consider either using a suitably modified RBE, or change the tolerance of key organs at risk in such situations. Recognition of changes of RBE with tissue and tumour α/β ratios, and appropriate use of these ratios to the clinical setting will be important in PBT.

**Figure 6:**
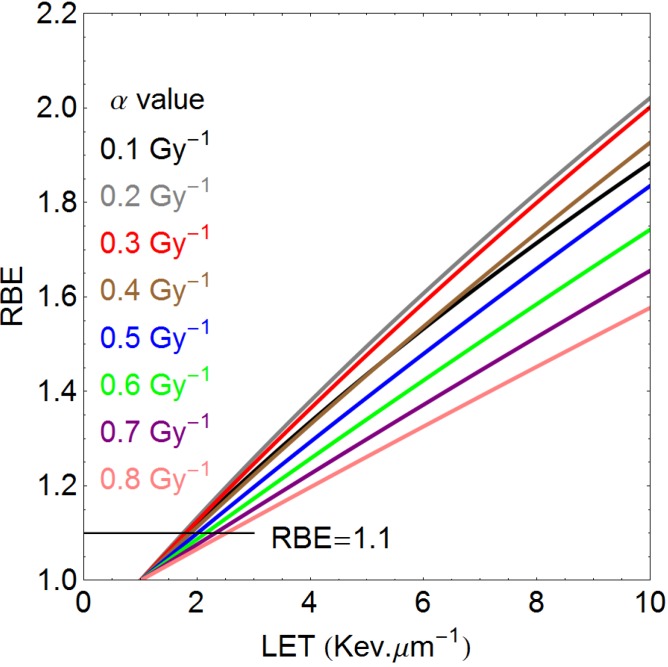
Relationship between LET and RBE, for variable low LET α values (Gy^−1^), as shown by the colour codes. The low LET β value is 0.035 Gy^−2^, increasing with LET. The change in the RBE rank order at lower values of α is due to the higher preponderance of β related cell kill at low α values.

Figure 6 shows the estimated effect of a variation in low LET α on the RBE values obtained with increasing LET, for a constant value of low LET β of 0.035 Gy^−2^. The rank order appears misplaced at lower α values, but this is a consequence of the relatively higher contribution of β-related cell kill in low α/β systems, so that RBE_min_ becomes more important and is approached at lower doses than would be the case for higher α/β systems. At the same time, cell systems which have low α values exhibit the highest RBE_max_ values but even at low dose (only 1.8 Gy of protons is used here) the overall RBE can be lower than for cell systems with higher α values if the ratio of α/β is small. Figure 6 must be interpreted by keeping Figure 5 in mind.

### 3.2. An “ICRU” Qualitative Matrix for Particle Beams

This is displayed in Table 1 where the up and down arrows indicate shifts in treatment dose allocation policies, or could be changes due dose delivery errors, with resultant effects on tumour cure probability (within PTV) and normal tissues complication probability (NTCP) which are relevant within and without the PTV. The rows exhibit different circumstances: row 1 (deliberate dose escalation/boost to tumour region), row 2 (more selective boost to gross tumour volume), row 3 (use of same tumour dose but reduction of collateral tissue dose, as in paediatric cancer), row 4 (unintended under-dosage of target periphery). In practice, greater complexity may be found, due to the use of several target volumes, each tailored to different expectations of tumour cell numbers with distance from a primary tumour, e.g., PTV_1_, PTV_2_, *etc.*

There are further uncertainties due to RBE allocation: if the RBE used is too low, then enhanced toxicity might occur in tissues which have higher RBE’s, but which are within the PTV; increased tumour recurrence rates could follow if the RBE has been overestimated for the tumour. These (not entirely straightforward) predictions may change in either direction (*i.e.*, better or worse)—if the dose delivery and/or assumed RBE is incorrect. For example, a shift of dose from one ICRU inner volume (GTV or CTV) category would result in tumour under-dosage but with over-dosage in an outer volume. Wherever misplaced Bragg peaks occur, regardless of cause, not only dose will change, but also LET and RBE. More research is required on such effects, with full use of the best available RBE allocation for all anatomical treatment sites and tumour types.

**Table 1:** Dose status for each ICRU volume and likely clinical outcome changes produced by CPT compared with MXT, where ↑ is an increased dose, = is the “equivalent” dose and ↓ is a reduced dose for the CPT. OTV is the volume of the body outside the PTV.

Dose Status	Tumour Control in GTV and {CTV+PTV}	{CTV+PTV} Side Effects	OTV Side Effects
GTV↑, {CTV+PTV}↑, OTV↓	Much better ***	Worse *	Better
GTV↑, {CTV+PTV}=, OTV↓	Better ***	Equal **	Better
GTV=, {CTV+PTV}=, OTV↓	Equal **	Equal **	Better
GTV=, {CTV+PTV}↓, OTV↓	Worse ****	Better	Better or worse

* In some instances reduced doses outside {CTV+PTV} may allow better radio-tolerance due to less late vascular insufficiency in the OTV. Also, this row condition may be acceptable in a non-essential tumour bearing tissue, or for a small volume of essential tissue with little risk of subsequent functional change. ** An equal outcome exists only if RBE is correct. *** In some instances (the radiosensitive tumour classes), tumour control will be very high with x-rays and there will be no gain in tumour control from dose escalation using any form of radiotherapy. These tumours may also have a lower RBE than 1.1, in which case the tumour control probability will be reduced if an RBE of 1.1 is used. **** Reductions in dose to CTV and PTV can occur due to range uncertainties and tissue movement, sometimes exacerbated by the use of scanned beams. The clinician may decide to extend the PTV and /or increase the local dose, depending on the tissue contained in the PTV and as a “trade-off” for gains in the OTV.

Such a simple qualitative, approach is helpful at the more basic level to assist teaching and strategic clinical thinking, but cannot give confident predictions, since changes in dose and LET produce non-linear effects in the cell killing processes that lead to clinical effects. However, some aspects are predictable: a shift in dose from GTV to other parts of PTV will be expected to reduce tumour control while enhancing normal tissue toxicity in tissues included within the PTV. A shift of dose from within the PTV to OTV will presumably always be associated with a reduced tumour control probability, while increasing dose and LET to OTV structures may or may not produce clinical effects depending on the radiation tolerance of the organ of risk and their RBE values.

This approach concentrates the mind in that, without knowing both LET and the true “biological dose” in the relevant volumes and associated organs at risk, then our knowledge and predictive abilities are incomplete. Then, it is possible that clinical results may disappoint compared to what was expected. It is essential that PBT clinicians should consider these issues. The added benefit of this approach would ideally be in the analysis of treatment results: all PBT treatment sites should be analysed using this ICRU framework with inclusion of realistic RBE assumptions (best and worst case scenarios), and tested for variations in intrinsic treatment delivery errors.

The future challenge will be to combine quantitative modelling with this categorical volume method. This would be useful in trial design and analysis since observed and expected results could then be compared in such a logical framework.

### 3.3. Two Clinical Examples Where PBT can be Suboptimal

In addition to the clinical example already given in Figure 2, the following two clinical entities are relevant.

#### 3.3.1. Prostate Cancer

Most American proton centres have based their business cases on treating high numbers of prostate cancer patients. There are some concerns about reports of enhanced side effects, but the outcomes are difficult to establish from small numbers in uncontrolled studies [35,36]. The following list of techniques and assumptions could contribute to enhanced toxicity and have been used in some/all treatment Centres:

(a) Use of one field per day treatments to save gantry sweep time, and only two field plans, which inevitably increase the dose per fraction outside the high dose treatment volume.
(b) Reduced beam shaping and conformity indices, especially with passive scattering, when compared to best photon techniques; this may be improved by scanned beams.
(c) Late complication RBE values are likely to exceed 1.1, so the biological doses to relevant volumes of small bowel and parts of rectum adjacent to the prostate can be higher than for photons.

It remains to be determined if better results emerge with scanned beams from all portals per day and using a higher RBE allocation of say 1.2 for normal tissues.

#### 3.3.2. Paediatric Cancers and Other Radiosensitive Tumours such as Lymphomas

**Figure 7:**
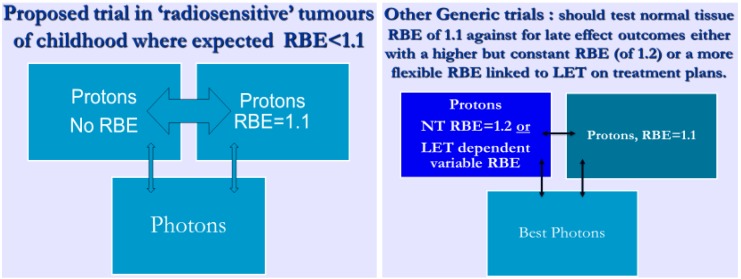
The proposal to randomise between a tumour RBE of 1 against a tumour RBE of 1.1 in radiosensitive tumours. Also, and in other studies of tumours of normal or reduced radiosensitivity, randomisation between an RBE of 1.2 (or higher) in late reacting normal tissues compared with 1.1 should also be considered.

Here, the tumour α radiosensitivity parameter is high, leading to low RBEs, sometimes less than the 1.1 used for protons [37]. In such cases, where RBEs may be as low as for example 1.03–1.07, the use of 1.05 or no RBE at all would respectively reduce or eliminate the chance of under-dosage. Such an approach would require careful assessment of the normal tissues close to the tumours, since they might then be overdosed, but in many cases of radiosensitive tumours this should be acceptable, since the relatively low curative doses are within normal tissue tolerance for severe late effects. Proposals for a clinical trial based on these concepts are shown diagrammatically in Figure 7. Random sampling simulations of tumour control probabilities, including numbers of patients required to reach significance are given elsewhere [38].

## 4. Discussion

Proton therapy remains the most promising external beam therapy method, but has some limitations. The obvious advantages of reduced or absent radiation exposure over wide tissue volumes will inevitably reduce many acute and late side effects. In the opinion of the present author, the physical and radiobiological uncertainties in tissues within and close to the target volumes could possibly be sufficient to cause unexpected late effects in some patients, by changing the delivered biological effective dose. Warnings regarding the RBE concerns have been made for many years [4,10,27], only to be ignored even by distinguished bodies such as ICRU [34]. Unless these important physical and radiobiological factors are together addressed, it is possible that proton therapy may give disappointing results, potential examples being slightly enhanced toxicity in tissues within or close to the PTV, and slightly reduced tumour control in some instances (if dose escalation beyond the photon range is not used). Some centres have published results which can be considered mildly disappointing [39,40], where spinal tumour relapses appear to be increased after protons, but in relatively small numbers of patients in retrospective publications of rare tumour treatments; such data are inconclusive. Few studies provide detailed study of late effects, one exception being the impressive maintenance of complex tissue function such as IQ and hearing [41]. A study from PSI in Switzerland attempts to correlate dose with temporal lobe brain necrosis without consideration that the RBE might be incorrect [42].

PBT centres should be given sufficient manpower resources and encouragement to publish comprehensive follow-up data on a regular basis and to cooperate nationally and internationally in order to build up larger data bases for analysis. Mandatory unexpected adverse radiation effect reporting must also be considered, as in the case of pharmaceutical products, where adverse events are reportable by law in most countries. A strong case can be made for a central world data collection for particle therapy, so that complications can be assessed with respect to Dose, LET and RBE. Such an approach would allow particle therapy to develop more safely and rapidly than in its present state of organisation. CERN, Geneva, with its data handling skills and particle physics expertise might be an ideal independent location for such work.

The new model for proton RBE determination uses independent α and β changes with LET. This essentially provides RBE estimations which depend on LET and the separate low LET α and β values, using a saturation function to relate these baseline radiosensitivities to the maximum radiosensitivities at the LET-RBE turnover points, allowing intermediate values of LET to scale the radiosensitivities with LET. Some published models do not use any change in β [31,43], along with empirically derived increments in α. Others have inputs of α/β [25,44] which should only be used for tentative modelling in low dose conditions. From the definitions of RBEmax and RBEmin, the relationship between RBEmax and α/β is inverse whereas the RBEmin will be directly proportional to the square root of α/β [31], but in the paper by Carabe *et al.* [25] two inverse functions are used. If RBEmin assumptions are incorrect then high dose effects be underestimated. The new model features allow α to increase more than β with LET, the α/β to increase with LET, but with independent changes in α and β that allow RBE max increase by more than RBEmin. The difference between RBEmax and RBEmin allow the cross over effects for different α/β values in the plots of RBE with dose per fraction. These cross overs predict a lower RBE at high dose per fraction for tissues with low α/β values (brain, normal tissue late effects). This is consistent with evolving practice in Japan towards hypofractionation because of fewer late side effects [45].

Considerable work needs to be done to improve treatment planning procedures, with rapid Monte Carlo simulations of beam transport and the production of 3-D LET maps which can be linked with cautious RBE predictions, to inform clinicians of the best achievable plans; dose maps alone with a constant RBE are insufficient [43,46]. More work needs to be done on the merits and disadvantages of scanned beams compared with passive scattering, with physical and radiobiological optimisation techniques for combinations of each, with assessment of using scanned beams for “boosts”, and to explore optimum combinations of spot size and beam weighting in order to produce the best overall biological dose conformity.

More basic research is required on the relationship between dose LET and RBE. A comprehensive recent review by Paganetti [38], including simulations, shows that higher RBE values can exist in parts of the beam. Further attempts to investigate this must be encouraged, as in the work of Cometto *et al*. [47], which attempts to bypass the RBE concept, but again excludes the potentially important increases in β with LET. Other authors (Heuskin *et al.*) [48] have used sophisticated Monte Carlo modelling including cell cycle progression, repair kinetics and bystander effect which are difficult to quantify and it is challenging to know how these may change with time during fractionated treatments.

Objections to non-human *in vivo* studies must consider the advantages of an intact organ system with its vasculature, even if the dose required to produce a clinically relevant outcome such as spinal paralysis is higher in small animals than in humans. However, the dose ratio of RBE is probably more robust than the absolute dose, thus minimising species effects.

Such essential basic research is necessary to provide more precise parameters for predictive modelling, since the data base available for all models that attempt to estimate RBE with increasing proton LET is far from comprehensive. The most reliable model would provide important information for clinical applications. Even if such a scientific programme might be considered “old science” these days, since it does not contain a molecular component, clinicians must insist on the future availability of such information for the sake of their patients. One interesting proposal is to establish a world research centre for proton and ion beam radiobiology and beam ballistics at CERN, Geneva, exactly for these and other purposes in order to extend basic science studies [49,50]. There, the RBE phenomenon could be comprehensively studied under standardised conditions, along with other cooperating institutes. The LET-RBE relationships must be investigated to greater precision, to determine accurate coefficients that control radiosensitivity changes with LET, their initial slopes and turnover point positions in a sensibly chosen panel of human cell lines and tissue systems capable of providing clinically applicable information. The available predictive models could then be compared. Such a centre could also become a world data collection system, with high quality analysis using the ICRU framework to provide radiation tolerance parameters in high LET treatments where unexpected adverse effects have occurred.

The above suggestions, along with more clinical research publications and molecular targeted approaches which might capitalise on the apparent reduction of RBE due to reduced repair capacity in some tumour types, as well as the better identification of RBE values in late reacting tissues, all of which might further improve proton therapy results.

It is also vital that proton therapy clinicians and physicists should understand the full implications of a switch between photon and proton therapy. This implies further detailed education of radiation oncology staff, rather than merely assuming that protons are a simple extension of conventional radiotherapy. It is clear that the large capital investments in PBT equipment should be accompanied by considerable further research and broader education of multidisciplinary staff involved in its delivery.

## 5. Conclusions

A more multidisciplinary approach is necessary to obtain optimum outcomes with proton therapy, integrating the clinical, physics and radiobiological aspects. Dose, LET and RBE needs to be computed and assigned to tissues, organs and tumours. Much more experimental work is required to determine RBE and its changes with LET and dose. The alternative is to press on regardless and adapt therapy doses to outcomes over a much longer time and after many disappointments.

## Figures and Tables

**Figure A2:**
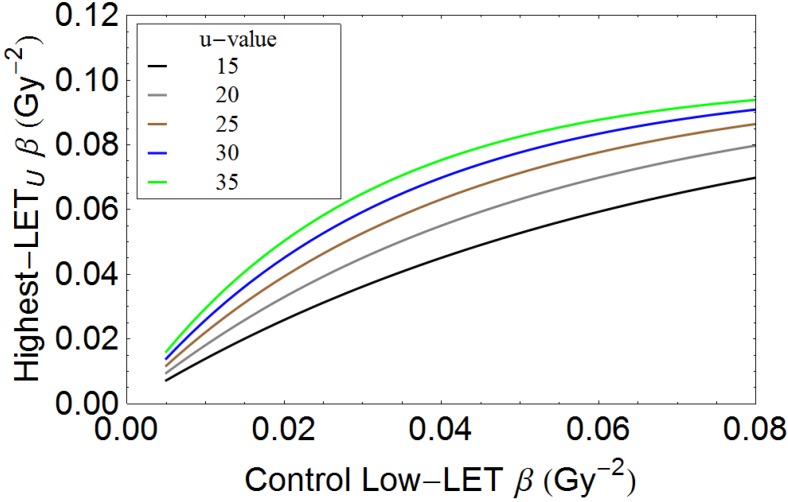
Plot of maximum value of β_U_ at LET-RBE turnover point and the control low LET β parameter, for variation in parameter u.

**Figure A3:**
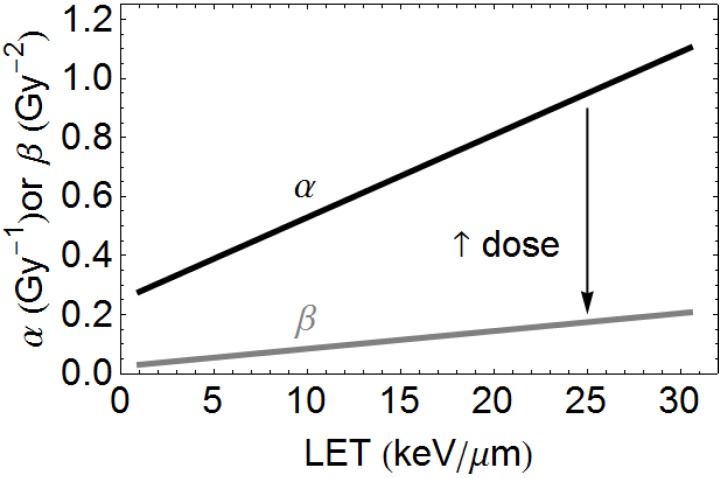
Schematic diagram of change in α and β with LET to show general principles for a hypothetical case. Increasing dose will increase the proportion of β mediated cell kill and so cause a lower overall RBE, as determined by both the increment in α (which changes RBE_max_) and in β (which change RBE_min_), and so the relative proportions of RBEmax and RBEmin which contribute to the overall RBE. The increase in β with LET is always smaller than that in α. In this way, with increasing dose, the overall RBE follows the direction of the arrow. The numbers used in this figure are arbitrary and not intended to represent any particular cell system.

## Appendix

The model considers increments in α and β in turn rather than assuming dependency of RBE with LET on the combined α/β ratio or on only α_L_. The relationship between the low LET control α_L_ and the maximum possible value of α (α_U_) at the turnover point of the LET-RBE relationship appears to be governed by a saturation process. Figure A1, taken from limited published data for ionic beams and protons [11,12,13,14,15,16] has the same overall form as more comprehensive data for fast neutrons, and can be fitted either a linear (for lower range α values) or over a longer range using a saturation function:

α_U_ = Q/j (1 – Exp(−j·α_L_))
(1)

with estimated values of Q = 10.62 and j = 3.92, from least squares fitting as shown in Figure A1.

This equation is based on the general case for a saturation process (by integrating the differential equation dα_U_/dα_L_= Q − j·α_U_, where Q is the initial gradient and j the saturation coefficient.

A similar “saturation” function, is used to link β_L_ with β_U_:

β_U_ = (R/u)·(1 – Exp(−u·β_L_))
(2)

Where R = 2.5 and u = 25, which provides a modest increase in β, chosen from Figure A2, compatible with the limited data discussed below and with a maximum ceiling value of 0.1 Gy^−2^ for β_U_.

When compared with α, the β parameter increases to a lesser extent with LET in human cell lines exposed to fast neutrons [27], which produce most of their ionisation by recoil proton collisions in hydrogen rich tissues. The carbon ion data of Weyrather *et al.* [16] show that β values rises from a control value of 0.026 Gy^−2^ to a maximum of 0.044 Gy^−2^ in V79 cells, and likewise from 0.02 to 0.042 Gy^−2^ for the CHO cells *i.e.*, by up to a factor of around two, which is small compared to the maximum increments in α with LET of around ten or more. The changes observed in V79 cells by Belli *et al*. [11] are inconclusive, as were the experiments of Chapman *et al.* using CHO cells [51]. Failure to include an increment of β with LET allows the RBE at high dose, referred to as RBE_Min_ [31,42], to be close to unity at higher doses. The increment in β must be symmetrical with that of α, since increasing doses produce symmetrical reductions in the LET-RBE plots [8,9,10], without any change in the turnover position; if β were to follow an alternative function with a different turnover point, this would not be so.

Since the proton LET-RBE turnover point (defined as LET_U_) is at 30.5 keV·µm^−1^ [11], and the increments in RBE (and consequently the radiosensitivity parameters) with LET appear to be linear [31,32], then with increasing dose the overall RBE will fall in proportion to the shift between the α and β parameters as shown in Figure A3.

Since α mediated cell kill predominates at low dose and β mediated cell kill at higher doses, it follows that the RBE will fall with increasing dose per fraction, by moving away from RBE_Max_ and closer towards RBE_Min_, where RBE_Max_ is the ratio α_H_/α_L_, and RBE_Min_ is the ratio √β_H_/√β_L_.

Consequently, the radiosensitivity values, α_H_ and β_H_, at any higher value of LET (LETx) than the control LET (LET_C_) will be, proportionately,

(3) $$\alpha_{H}=\alpha_{L}+\frac{LETx-LET_{C}}{LET_{U}-LETc}\cdot \left(\alpha_{U}-\alpha_{L}\right)$$

and

(4) $$\beta_{H}=\beta_{L}+\frac{LETx-LET_{C}}{LET_{U}-LETc}\cdot \left(\beta_{U}-\beta_{L}\right)$$

LET, expressed as a fraction of the LET_U_, then reflects the efficiency of the cell killing, changing the cell kill coefficients α and β.

The overall RBE is given by the solution of an isoffect equation between the control and the high LET, as in

α_L_·d_L_ + β_L_·d_L_^2^ = α_H_·d_H_ + β_H_·d_H_^2^ (5)

The quadratic solution for d_L_ is then divided by d_H_ to provide the RBE [20,28].

Biological effective dose equations [27,52] for both low (control) LET and higher LETs, both contain the low LET α/β ratio. The two RBE limits are governed by the equations given above, for the isoeffect:

(6) $$d_{L}\left(1+\frac{d_{L}}{{\left(\frac{\alpha }{\beta }\right)}_{L}}\right)=d_{H}\left(RBE_{Max}+\;\frac{RBE_{Min}^{2}\cdot \;d_{H}}{{\left(\frac{\alpha }{\beta }\right)}_{L}}\right)$$

Values of RBE_Max_ and RBE_Min_ can be obtained using the estimated α_H_ and β_H_ results; the low LET α/β is known, and so RBE can be obtained by solution of the quadratic equation for d_L_ in Equation (6), and dividing the result by d_H_.
